# Evaluation of Physicochemical and Mechanical Properties of a Modified Adhesive System by Resveratrol Incorporation

**DOI:** 10.3390/jfb16050178

**Published:** 2025-05-14

**Authors:** Amanda Guedes Nogueira Matuda, Karen Cristina Kazue Yui, Nathália Moreira Gomes, Gabriela da Silva Chagas, Marcella Batista Rocha, Fernanda Labiapari Senefonte, Mariane Cintra Mailart, Cesar Rogério Pucci

**Affiliations:** 1 Department of Restorative Dentistry, Institute of Science and Technology, São Paulo State University (UNESP), São José dos Campos 12245-000, SP, Brazil; amanda.matuda@unesp.br (A.G.N.M.); nathalia.m.gomes@unesp.br (N.M.G.); gabriela.chagas@unesp.br (G.d.S.C.); marcella.b.rocha@unesp.br (M.B.R.); fernanda.labiapari@unesp.br (F.L.S.); mariane.cintra-mailart@unesp.br (M.C.M.); 2 Department of Pediatric and Social Dentistry, Institute of Science and Technology, São Paulo State University (UNESP), São José dos Campos 12245-000, SP, Brazil; karen.yui@unesp.br

**Keywords:** anti-bacterial agents, bioactive materials, dentin-bonding agents, mechanical tests, resveratrol

## Abstract

This study aimed to evaluate the physicochemical and mechanical properties of a modified adhesive system containing resveratrol by assessing its microtensile bond strength (µTBS), degree of conversion (DC), mini-flexural strength (MFS), and antibacterial activity. The modified etch-and-rinse adhesive system was prepared by resveratrol (RES) incorporation in different concentrations: adhesive with 0.5% RES (RES0.5), adhesive with 1% RES (RES1), adhesive with 2% RES (RES2), and adhesive with no RES incorporation (RES0—control group). The µTBS test was conducted on 40 human molars with dentin exposure, which were etched, bonded with the adhesives (*n* = 10), and restored with resin composite. Fourier Transform Infrared Spectroscopy (FTIR) measured the DC for the MFS; ten adhesive sticks were made for each group. Antibacterial activity was assessed using colony-forming unit (CFU) counts. For µTBS, no difference between the groups was found (mean ± SD): RES0.5—42.93 ± 15.49^A^; RES1—42.61 ± 13.97^A^ and RES2—39.43 ± 9.14^A^; RES0—41.01 ± 2.64^A^. The DC (% ± SD) of the experimental groups was similar: RES0.5—81.02 ± 1.95^A^; RES1—76.02 ± 9.00^A^; RES2—58.86 ± 15.94^A^; RES0—77.75 ± 3.22^A^. For MFS (mean ± SD): RES0.5—33.14 ± 13.83^A^; RES1—31.1 ± 12.21^A^; RES2—19.72 ± 5.43^B^; RES0—29.72 ± 11.95^A^. For CFU (mean ± SD): RES0.5—0.67 × 10^7^ ± 0.37^B^; RES1—0.68 × 10^7^ ± 0.34^B^; RES2—0.60 × 10^7^ ± 0.02^C^; RES0—0.75 × 10^7^ ± 0.03^A^. The incorporation of resveratrol into the adhesive system at low concentrations (0.5 and 1%) does not alter the bond strength of the adhesive interface, the degree of conversion, or the flexural strength. Additionally, both concentrations exhibited antibacterial properties by reducing the colony-forming units of *S. mutans*.

## 1. Introduction

The clinical performance of restorations is fundamental for the longevity of treatments. However, in both anterior and posterior teeth, failures might lead to the repair or replacement of restorations, depending on the clinician’s diagnosis, as well as the type and extension of failure. The replacement of resin composite restorations is a common procedure in daily clinical practice, leading to larger and more complex restorations [1]. In general, the main reasons for resin composite restoration failure are compromised esthetics, fractures, and secondary caries [2], which is the most prevalent cause of replacement of dental restorations. Secondary caries occurs due to the penetration of bacteria and their by-products through the adhesive interface, leading to its degradation [3,4]. To address this issue, one of the essential aspects of the restorative procedure is to ensure that the adhesive systems are properly made to enhance the durability of the tooth–adhesive interface, in addition to providing instructions for oral hygiene and ensuring patient compliance. Secondary caries and adhesive interface degradation represent relevant factors in the longevity of resin–dentin bonds [5,6,7].

To prevent the degradation of the dentin–adhesive interface, it is required that the adhesive system fill the spaces created around the demineralized collagen fibrils. If this is not achieved, a weakened zone is formed within the hybrid layer, leading to nanoleakage, hydrolysis of collagen fibrils, and, ultimately, degradation of the adhesive interface [1,8]. As a consequence of the degradation, a reduction in the bond strength between the tooth and the restoration occurs. Furthermore, this compromises the ability to seal the cavity, potentially resulting in marginal staining, secondary caries, and premature loss of both direct and indirect restorations [9].

The incorporation of bioactive molecules with antimicrobial or remineralizing capabilities into adhesive systems has demonstrated the potential to enhance mechanical strength and inhibit the enzymatic degradation of the adhesive interface, thereby prolonging their durability [10]. In this context, a bioactive molecule with antioxidant properties can also be favorable, since this is a substance that significantly delays or inhibits the oxidation of an oxidizable substrate [11] by suppressing oxidative stress and also reduces metalloproteinase activity by inhibiting free radicals [12]. It is established that the incorporation of antioxidants into adhesive systems stabilizes and increases the number of collagen cross-links, while simultaneously reducing the hydrolysis of collagen fibrils and the degradation of the hybrid layer [13]. Among the distinct antioxidants, polyphenols are widely present in nature and can be found in various foods and beverages, including fruits, vegetables, tea, wine, and coffee [14]. These agents have the potential to enhance collagen network stability by promoting an increase in cross-link formation between collagen fibrils while simultaneously reducing collagen enzymatic degradation [12]. Polyphenols have been incorporated in different materials to enhance adhesion in dentin, and promising results have been achieved, such as decreased enzymatic degradation, reduced swelling ratio of demineralized dentin, and increased strength [15,16].

Specifically, the polyphenol-rich grape seed extract enhances the mechanical properties of demineralized dentin, including modulus of elasticity and tensile strength [17,18], and promotes chemical modification to the dentin matrix, resulting in increased bond strength of the dentin–resin interface [19]. Another polyphenol present in grapes, as well as peanuts and cranberries, is resveratrol (3,5,4′-trihydroxy-trans-stilbene). It is a member of the stilbene family and a precursor to other stilbenes, including viniferins and pterostilbene [20,21]. Resveratrol has many benefits, such as protective potential against oxidative damage, protecting oral fibroblasts from reactive oxygen species inducers, inhibiting MMP activity, promoting biomimetic remineralization [14,22,23,24], inhibiting the physiological activity of cariogenic bacteria, reducing acid production and tolerance, and inhibiting the synthesis of polysaccharides, thus compromising the formation of biofilm and subsequently, preventing caries [21,25].

Although the benefits of resveratrol are well-reported in the literature, there is limited evidence regarding its potential effects when incorporated into the adhesive system for dentin adhesion. Therefore, evaluating a promising component like resveratrol, not only in terms of bonding but also preserving the adhesive interface, could be crucial for improving the longevity of adhesive restorations [26]. Thus, the objective of this study was to evaluate the physicochemical and mechanical properties of a modified adhesive system containing resveratrol by assessing its microtensile bond strength, degree of conversion, mini-flexural strength, and antibacterial activity. The null hypotheses test whether there is no difference in incorporating distinct concentrations of resveratrol in an etch-and-rinse adhesive system regarding: H01—the microtensile bond strength (µTBS) of the adhesive interface; H02—the degree of conversion (DC) of the adhesive; H03—the mini-flexural strength (MFS) of the adhesive; H04—antibacterial activity.

## 2. Materials and Methods

### 2.1. Ethical Aspects

Extracted sound human molars (*n* = 40) were obtained from patients at the University who provided informed consent to donate their teeth for research purposes. The study protocol was submitted to and approved by the Local Research Ethics Committee (protocol: #02035118.1.0000.0077).

### 2.2. Study Design

This in vitro study included four experimental groups, according to different concentrations of resveratrol incorporated into an etch-and-rinse adhesive system: the commercial adhesive with 0.5% resveratrol (RES0.5), the commercial adhesive with 1% resveratrol (RES1), the commercial adhesive with 2% resveratrol (RES2), and the commercial adhesive with no incorporation (RES0). The response variables analyzed included microtensile bond strength (µTBS), degree of conversion (DC), mini-flexural strength (MFS), and antibacterial activity, which were assessed using colony-forming unit (CFU) analysis.

### 2.3. Preparation of Adhesive System

The concentrations of resveratrol (Sigma-Aldrich, St. Louis, MO, USA) incorporated into the conventional adhesive system Adper Single Bond 2 (3M ESPE, St. Paul, MN, USA) were obtained by weighing on a precision balance (Mettler-Toledo, Greifensee, CH, Switzerland), and the mixture was carried out in amber-colored flasks for each group. The flasks were placed on the balance and tared before adding the adhesive. After obtaining the weight of the adhesive, the weights equivalent to each percentage of resveratrol were calculated to proceed with the incorporation. The resveratrol powder was added to the adhesive at the following concentrations: 0.5 wt%, 1 wt%, and 2 wt%. To ensure a homogeneous experimental product, the resveratrol and adhesive were mixed using an asymmetric double centrifugal mixer (SpeedMixer DAC 150.1, FVZ, FlackTek, Landrum, SC, USA) for 5 min at a speed of 3500 rpm. Afterward, the flasks were kept under constant agitation (Orbit 300, LabNET International Inc., Edison, NJ, USA) for 48 h, and then, they were stored in dark bottles under refrigeration (4 °C). Prior to use, they were mixed again. Table 1 describes the composition of the adhesive system and resveratrol used in this study.

### 2.4. Microtensile Bond Strength (µTBS)

The G*Power 3.1 program was used to calculate the sample size, based on pilot data. The results were entered into the G*Power 3.1 program, where the effects of F of 1.25, α of 0.05, and β of 0.8, and the number of groups of 2 were obtained. The critical value of F was 5.98, and the total number of specimens was 8, with a power of 0.83. To ensure data robustness and accommodate possible specimen loss, the study was performed with 10 specimens per group, and the same operator was standardized to carry out all stages of the test.

Forty sound extracted human molars were selected and taken to a precision cutting machine (Labcut, Extec; Enfield, CT, USA) with abundant water cooling to remove the enamel surface, resulting in flat surfaces with exposure of the coronal dentin. The dentin surfaces were polished with 600-grit silicon carbide water sandpaper in a polishing machine under cooled water at 300 rpm for 30 s to create a standardized smear layer [27,28].

After polishing procedures, the dentin surface was conditioned for 15 s with 37% phosphoric acid gel. Afterward, the surface was rinsed with distilled water for 30 s and carefully dried with absorbent paper to maintain the moisture of the dentin surface [29]. Then, the adhesive system was actively applied with a disposable applicator (Microbrush, KG Sorensen, Barueri, SP, Brazil) for 20 s, according to the different concentrations of resveratrol (*n* = 10). Two layers of adhesive were applied and dried with air for 10 s. Then, it was light-cured (Radii-cal, SDI, Bayswater, VIC, Australia) for 20 s with 1200 mW/cm^2^. Two 2 mm thick increments of resin composite (Filtek Z350, 3M ESPE, St. Paul, MN, USA) were added and individually light-cured for 40 s, leading to 4 mm thick restoration. The restored teeth were stored in distilled water at 37 °C for 24 h.

The restored teeth were sectioned in a precision cutting machine at low speed under water cooling, parallel to the long axis of the tooth to obtain 1 mm thick sticks containing composite resin, adhesive interface, and dentin. The sticks from the edges were discarded, and six sticks were selected from each group to be submitted to the test. The sticks were attached to a metallic microtensile device in a universal testing machine (DL-200, EMIC, Sao Jose dos Pinhais, PR, Brazil). Each stick was tested in tension until failure at a crosshead speed of 0.5 mm/min using a 10 kgf load cell, according to the International Standards Organization 11405 Standard [30]. The bond strength data were expressed in megapascals (MPa).

At the end of the test, the fractured parts were cleaned, dried, and analyzed for the fracture pattern using an optical stereomicroscope with 40× magnification (Zeiss, steREO Discovery V20, Göttingen, Lower Saxony, Germany). The failure modes were classified according to the type of fracture, such as adhesive, cohesive in resin, cohesive in the dental structure, and mixed. Premature and cohesive failures were not considered in the statistical analysis.

### 2.5. Degree of Conversion (DC)

To measure the degree of conversion, a Fourier Transform Infrared Spectrometer (FTIR, Spectrum 400; Perkin-Elmer, Waltham, MA, USA) was used, with a resolution of 4 cm^−1^ in Attenuated Total Reflection (ATR) sampling mode. A 10 μL drop of each adhesive (*n* = 3) was deposited on the ATR crystal, covered with a transparent coverslip, and fixed with adhesive tape to prevent evaporation of the adhesive components. The adhesive was then light-cured (Radii-cal, 1200 mW/cm^2^) for 20 s. A time-resolved spectrum collector (Spectrum TimeBase, Perkin-Elmer, Waltham, MA, USA) was used for continuous and automatic collection of spectra during polymerization [31,32].

The spectral range from 4000 to 650 cm^−1^ was used to obtain the spectra of the uncured adhesive and the corresponding polymerized adhesive. A change in the band height ratios of the aliphatic carbon–carbon double bond (peak at 1638 cm^−1^) and aromatic C=C (phenyl peak at 1608 cm^−1^) was observed in the cured and uncured states. To calculate the degree of conversion (DC), the formula based on the decrease in the intensity of the band ratios before and after light curing was used (Equation (1)).

(1) $$\mathrm{DC}=100\;\times \;(1-\frac{Absorbance\;after}{Absorbance\;before})$$

### 2.6. Mini-Flexural Strength (MFS)

Specimens of the adhesive system for each experimental group (*n* = 10) were obtained using silicone matrices with dimensions of 12 mm × 2 mm × 2 mm (Odeme Equipamentos Medicos e Odontologicos Ltda., Luzerna, SC, Brazil). The adhesive system was applied to the matrices with a micropipette and light-cured (1200 mW/cm^2^) for 40 s. The specimens were placed in individual amber bottles for subsequent analysis.

To evaluate flexural strength, the specimens were positioned in metallic devices and submitted to a flexural force in a universal testing machine (DL-200, EMIC, Sao Jose dos Pinhais, PR, Brazil). A 10 kg load cell was used, as well as a metal pin at a speed of 0.5 mm/min to apply a 0.5 Nm/s force in the central portion of the specimen. The distance between the support points was carefully set at 9.0 mm.

The results were expressed in MPa, using Equation (2), where σ is the flexural strength (in MPa), F is the maximum load at fracture (N), I is the length between the support points (9 mm), b is the width of the adhesive specimen (2 mm), and h is the thickness of the adhesive specimen (2 mm).

(2) $$\sigma =3FI/2bh^{2}$$

### 2.7. Antibacterial Activity

To test the antibacterial activity, Streptococcus mutans derived from carious dentin (ATCC 25175, American Type Culture Collection, Manassas, VA, USA) was used. This strain was cultivated aerobically in Brain Heart Infusion (BHI; MilliporeSigma, St. Louis, MO, USA) broth at 37 °C. After overnight growth, the bacterial cells were harvested, washed three times using sterile phosphate-buffered saline, and resuspended in BHI to a final concentration of 1.0 × 10^7^ colony-forming units (CFU)/mL. The bacterial density was quantified spectrophotometrically (Beckman Coulter, Inc., Indianapolis, IN, USA) at 600 nm [6].

A Drigalski spatula was used to spread bacterial suspensions (1.0 × 10^7^ CFU/mL) onto BHI agar plates. Disks of polymerized adhesive resin (6.5 mm diameter × 1.5 mm thickness) were prepared according to the groups and were individually placed on the bacterial-inoculated agar plates. These plates were then incubated at a temperature of 37 °C for 24 h.

To determine the colony-forming units (CFUs), the adhesive disks were carefully transferred to BHI agar plates. Following the incubation period, a 150 µL aliquot of the supernatant was collected. The optical density of the collected samples was measured using a spectrophotometer (Spectra Count, Packard Instrument Co., Meriden, CT, USA) at 600 nm. Bacterial viability was assessed by counting the CFUs. The entire procedure was repeated in triplicate.

### 2.8. Statistical Analysis

Quantitative data obtained for the μTBS, degree of conversion, mini-flexural strength, and CFU counts were analyzed by a blinded operator using Statistica for Windows (Data Analysis Software System, Version 8.0). They were tested for their normality (Shapiro–Wilk test, *p* = 0.05), then separately analyzed using one-way ANOVA followed by the post-hoc Tukey’s test. The statistical significance level was pre-set at α = 0.05.

## 3. Results

Regarding the microtensile bond strength (µTBS), no significant differences between the experimental groups were found (*p* > 0.05) (Table 2). The incorporation of resveratrol exhibited no effect on microtensile bond strength.

**Figure 1:**
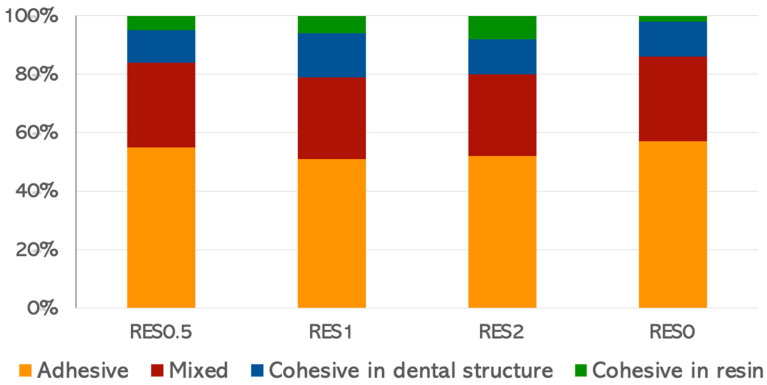
Percentage of fracture types after the microtensile bond strength test.

Only adhesive and mixed fracture types were considered for the results, and there was a predominance of mixed and adhesive failures over cohesive failure patterns (Figure 1).

**Figure 2:**
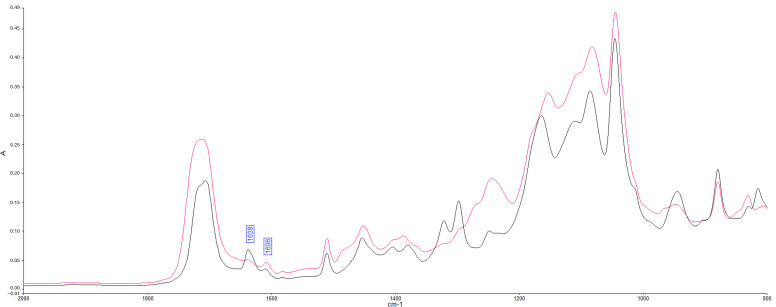
Representative FTIR spectra of the adhesive before (black line) and after (pink line) light activation, showing the characteristic peaks of aliphatic (1638 cm^−1^) and aromatic (1608 cm^−1^) C=C bonds. The reduction in the aliphatic band after light curing indicates monomer conversion. A = absorbance; cm^−1^ = wavenumber.

The presence of resveratrol at concentrations of 0.5 wt%, 1 wt%, and 2 wt% exhibited no significant effect on the degree of conversion of the adhesive system, since they were similar to the control group (RES0) (*p* > 0.05) (Table 2, Figure 2).

Regarding the mini-flexural strength (MFS), the lowest value was found for the RES2 group (2 wt% resveratrol). Similar MFS values were found for the RES0.5, RES1, and RES0 groups (*p* < 0.05) (Table 2).

For CFU counts of *S. mutans*, the adhesive disks containing 0.5 wt%, 1 wt%, and 2 wt% resveratrol presented significantly lower viable bacteria compared to the control adhesive (RES0) (*p* < 0.05). The concentrations of 0.5 wt% and 1 wt% were similar, and the lowest value of CFU was found for the RES2 group (2 wt%) (Table 2).

## 4. Discussion

The results of µTBS demonstrated that the incorporation of resveratrol into an etch-and-rinse adhesive system did not interfere with the bond strength of the adhesive interface, thus, the first hypothesis was not rejected. The µTBS test was first described by Sano et al. (1994) [33], and it is extensively reported in the literature. In this study, the commercial conventional adhesive Adper Single Bond 2 was chosen due to its great performance in other studies [34,35,36,37,38]. Through a pilot study, the low concentrations of resveratrol, such as 0.5, 1, and 2%, were tested and defined to be used. Additionally, regarding resveratrol incorporation into the adhesive system, it was previously reported that 0.1 and 1 mg/mL concentrations did not affect the bond strength and also improved the durability of adhesion [39]. However, it is important to highlight that appropriate concentrations of resveratrol must be carefully determined to ensure that its inclusion in adhesives minimally affects adhesive polymerization, bond strength, and bond durability [39], as observed in our study.

The impact of adding resveratrol (0.5 μM) to distinct adhesive systems has previously been investigated in terms of micro-tensile bond strength and cytotoxicity [20]. The reported bond strength findings are in accordance with those of this study, since the resveratrol addition did not show any negative effect in micro-tensile bond strength to dentin. Additionally, resveratrol incorporation also enhanced cell viability. However, there were differences between the groups, considering the type of adhesive as a variable [20]. HEMA-free and UDMA-based adhesive systems exhibited lower μTBS values, which lends support to the hypothesis that the absence of HEMA results in water contact with hydrophobic groups, thereby creating unfavorable conditions that cause water separation [40]. Thus, the interaction of resveratrol with different combinations of monomers results in distinct effects, as different monomers present varying diffusion properties, which play a key role in adhesion [20,39,41]. In this study, a total-etch adhesive was used due to the absence of acidic monomers, such as 4-META, in its composition. Additionally, the presence of HEMA in its formulation supported the results obtained.

The results of the degree of conversion demonstrated that none of the resveratrol concentrations altered the degree of conversion values, thus leading to not rejecting the second hypothesis. Lower concentrations of resveratrol, such as 0.1 and 1 mg/mL, have been shown not to significantly decrease the degree of conversion, behaving similarly to a control group of a commercial universal adhesive system without resveratrol incorporation [39]. In contrast with these groups, for the experimental adhesive system with 10 mg/mL resveratrol, a significant decrease was observed. The incorporation of resveratrol in adhesive systems should be carefully investigated to achieve optimal bond strength without compromising the degree of conversion.

The degree of conversion is defined as the ratio of the double bond content of the monomer to the polymer, calculated as the ratio of C=C double bonds in cured and uncured materials. It is rare for this conversion to be complete, but it is essential to find high values for it, since a low degree of conversion is associated with a decline in physicochemical properties and a compromised biocompatibility, since residual monomers can diffuse into the pulp or even elute into the oral cavity [36,42,43]. Although there is no standard acceptable value for the degree of conversion, values close to 85% for TEGDMA and 55–65% for TEGDMA/Bis-GMA blends can be found [44]. More specifically, average values of 78% [45], 82% [46], and 84% [36] have already been reported in the literature for the adhesive system used in this study, obtained with different methodologies, such as FT-MIR, FT-NIRS, and micro-Raman spectroscopy, respectively. The degree of conversion may be influenced by the light-curing device used [45] and by the viscosity of the adhesive, in which the lower the viscosity, the higher the degree of conversion [43]. Furthermore, the mechanical properties of resin materials are directly affected by the cross-linking density and the quality of the network that forms during polymerization [47]. In this context, it is possible to speculate that the higher concentration of resveratrol in the RES2 group increased the viscosity of the experimental adhesive or might have impaired the cross-linking of the polymer matrix, which may also be associated with the decline in mini-flexural strength.

While 0.5% and 1% resveratrol did not significantly impact the mini-flexural strength values, the 2% resveratrol resulted in a notable decline in mini-flexural resistance; thereby, the third hypothesis was rejected. Negative effects on the mechanical properties of resin-based materials can be observed after the addition of fillers due to a significant reduction in inter-particle spacing, increasing the number of particle collisions and impairing suspension viscosity, which may lead to a decrease in dimensional stability, bond strength, and flexural strength [48]. Flexural strength is a mechanical property that refers to the resistance to failure caused by tension in the resin material, measured by its curvature [49], and is directly affected by the final conversion after light curing [50]. Although numerical results alone are insufficient to predict the clinical behavior without considering individual factors, adhesive restorations are constantly susceptible to considerable flexural and functional tension forces [51]. Therefore, it is expected that experimental materials should either improve or at least maintain the tested properties compared to control groups.

There is limited literature on the impact of resveratrol on the mechanical properties of adhesive systems. Although some studies have tested the effect of resveratrol and other polyphenols on the bonding properties, there is a scarcity of investigations regarding the flexural strength. However, the incorporation of resveratrol was tested in orthodontic acrylic resin for estimating flexural strength [52], and the dose-dependent impact of resveratrol was also verified for this property. The study [52] showed that modified poly (methyl methacrylate) with resveratrol is a viable option as an antimicrobial acrylic resin for clinical use. Then, the differential of this study is to analyze the effect of distinct low concentrations of resveratrol in mini-flexural resistance.

The incorporation of different concentrations of resveratrol (0.5, 1, and 2%) to the adhesive significantly reduced viable bacteria compared to the control group, and the 2% resveratrol demonstrated a better antimicrobial activity effect. Then, the fourth null hypothesis was rejected. Also, considering that the 2% concentration showed better results compared to the others, but presented a loss in flexural strength, it may be interesting to test intermediate concentrations between 1% and 2% in the next studies.

Cariogenic bacteria, such as *S. mutans*, possibly lead to demineralization and degradation of the dentin tissue, compromising the integrity of adhesive restorations [53]. However, it has been shown [21,54] that resveratrol is capable of restraining the growth of Gram-positive bacteria, such as *S. mutans*, and it can also alter the bacterial virulence, which contributes to a reduction in the synthesis of toxic substances and inhibition of biofilm formation.

Although no studies with methodologies that allow direct comparison of results have been found to date, literature reports [55] describe evaluations using confocal laser microscopy and mitochondrial activity (MTT assay) to assess the inhibition effect of live bacteria on dentin slices pretreated with resveratrol/ethanol solution. These studies confirm that the solution inhibited the growth of *S. mutans* in the dentin substrate. Caution is necessary to extrapolate the results since the substance was applied as a pretreatment and was not incorporated into the adhesive system; however, the solution showed potential to protect against recurrent caries and also improved the durability of dentin bonding. Resveratrol demonstrates a marked inhibitory potential against *S. mutans*, revealing an anticaries potential [56], which reinforces the interest in adding it to the adhesive system. It is also known that its antibacterial effect against *S. mutans* is concentration-dependent and shows both bacteriostatic and bactericidal properties [57]. This indicates that the antimicrobial activity of resveratrol can be modified by altering its concentration, which may have potential applications in dental health.

Resveratrol may enhance the durability of the dentin bond, inhibit the formation of *S. mutans* biofilms, and consequently contribute to reducing the incidence of secondary caries [55]. Antibacterial properties and biofilm inhibition are essential characteristics of adhesive systems, resulting in the longevity of restorations. The present study yielded consistent results from the CFU assay, demonstrating that *S. mutans* biofilm formation was significantly lower at varying resveratrol concentrations. Therefore, resveratrol may have an effective inhibitory capacity in preventing secondary caries in a clinical condition.

However, caution is required when interpreting the findings of this study since only the two-step etch-and-rinse adhesive system was tested. Additionally, authors [58] suggest that adhesive bond strength may also be influenced by aging conditions and simulated pulp pressure in laboratory settings. Furthermore, our experimental design focused solely on CFU counts after incubation with the adhesive disks. As a result, we cannot determine whether the antibacterial effect was due to resveratrol elution or direct contact with the solid adhesive surface. Future studies should explore these points to better predict the durability of dentin restorations under clinical conditions.

## 5. Conclusions

The incorporation of resveratrol into an adhesive system at low concentrations (0.5 and 1%) does not alter the bond strength of the adhesive interface, the degree of conversion, or the flexural strength. Additionally, both concentrations exhibited antibacterial properties by reducing the colony-forming units of *S. mutans*.

## Figures and Tables

**Table 1:** Composition of the adhesive system and resveratrol used in the study, according to manufacturers’ specifications.

Product	Manufacturer	Composition	Batch Number
Adper Single Bond 2	3M ESPE, St. Paul, MN, USA	Bisphenol-A-glycidyl-dimethacrylate, 2-hydroxylethyl methacrylate, dimethacrylate, water, ethanol, photoinitiator, silica nanoparticles, methacrylate functional copolymer of polyacrylic and polyalkenoic acids	2129900256
Resveratrol	Sigma-Aldrich, St. Louis, MO, USA	3,4′,5-Tri-hidroxi-trans-estilbeno 5-[(1E)-2-(4-hidroxifenil) etenil]-1,3-benzenodiol	SLBV8562

**Table 2:** Mean values and standard deviations (SD) of μTBS values (MPa), % degree of conversion (DC), MFS (MPa), and recovered *S. mutans* (CFUs).

Groups	µTBS (±SD) *	% DC (±SD)	MFS (±SD) *	CFUs (±SD)
RES0	41.01 (±2.64) A	77.75 (±3.22) A	29.72 (±11.95) A	0.75 × 10^7^ (±0.03) A
RES0.5	42.93 (±15.49) A	81.02 (±1.95) A	33.14 (±13.83) A	0.67 × 10^7^ (±0.37) B
RES1	42.61 (±13.97) A	76.02 (±9.00) A	31.1 (±12.21) A	0.68 × 10^7^ (±0.34) B
RES2	39.43 (±9.14) A	58.86 (±15.94) A	19.72 (±5.43) B	0.60 × 10^7^ (±0.02) C

* = values expressed in MPa. Different uppercase letters indicate significant differences between the experimental groups for each response variable (differences in columns) (*p* < 0.05, Tukey’s test).

## Data Availability

The original contributions presented in the study are included in the article, further inquiries can be directed to the corresponding author.
